# Bariatric surgery in individuals with human immunodeficiency virus and type 2 diabetes: a case series

**DOI:** 10.1186/s13256-019-2078-8

**Published:** 2019-05-10

**Authors:** Wei Yang, Anjali Zalin, Mark Nelson, Gianluca Bonanomi, James Smellie, Kevin Shotliff, Evangelos Efthimiou, Veronica Greener

**Affiliations:** 1 Bariatric Medicine, Chelsea and Westminster Hospital, 369 Fulham Road, Chelsea, London, SW10 9NH UK; 2 Diabetes and Endocrinology, Diabetes and Metabolism, Chelsea and Westminster Hospital, 369 Fulham Road, Chelsea, London, SW10 9NH UK; 3 HIV Medicine, Chelsea and Westminster Hospital, 369 Fulham Road, Chelsea, London, SW10 9NH UK; 4 Bariatric Surgery, Chelsea and Westminster Hospital, 369 Fulham Road, Chelsea, London, SW10 9NH UK; 5 Endocrine and Thyroid Surgery, Chelsea and Westminster Hospital, 369 Fulham Road, Chelsea, London, SW10 9NH UK; 6 Endocrinology and Bariatric Medicine, Chelsea and Westminster Hospital, 369 Fulham Road, Chelsea, London, SW10 9NH UK; 7 Beta Cell Diabetes Centre, Chelsea and Westminster Hospital, 369 Fulham Road, Chelsea, London, SW10 9NH UK

**Keywords:** Bariatric surgery, Type 2 diabetes, Human immunodeficiency virus

## Abstract

**Background:**

The efficacy and safety of bariatric surgery have not been fully elucidated in patients affected with human immunodeficiency virus. Although adjustable gastric banding and sleeve gastrectomy are starting to be used in patients with human immunodeficiency virus, there are limited descriptions of the outcomes of type 2 diabetes mellitus in individuals who are human immunodeficiency virus positive and undergoing these procedures.

**Case presentation:**

We have evaluated retrospectively three patients who underwent adjustable gastric banding or sleeve gastrectomy, the effect in weight reduction and glycemic control as well as its impact on human immunodeficiency virus management. Case 1 (adjustable gastric banding), a 58-year-old Caucasian male, achieved 19% total weight loss, Case 2, a 33-year-old Caucasian male (sleeve gastrectomy) lost 25%, and Case 3, a 48-year-old Caucasian female (sleeve gastrectomy), lost 14% postoperation. In terms of type 2 diabetes mellitus, Case 2 achieved complete remission according to American Diabetes Association criteria, while Case 1 would also have achieved remission were it not for the continuation of metformin postoperatively. Insulin requirements and pill burden were markedly reduced in Case 3 after sleeve gastrectomy, although lack of remission was predictable given the longevity of type 2 diabetes mellitus and preoperative insulin dosage. In all three cases, human immunodeficiency virus status did not appear to be affected by the bariatric surgery which was supported by the postoperative stable CD4 count and undetectable viral load.

**Conclusions:**

Bariatric surgery is a safe and effective treatment modality in patients who are human immunodeficiency virus positive with obesity and type 2 diabetes mellitus.

## Background

Obesity, type 2 diabetes mellitus (T2DM), and human immunodeficiency virus (HIV) are prominent global health issues. With the advent of highly active antiretroviral treatment (HAART) and improved mortality rates, people with HIV infection increasingly present with obesity and related metabolic consequences [1]. Bariatric procedures, including adjustable gastric banding (AGB), sleeve gastrectomy (SG), and Roux-en-Y gastric bypass (RYGB), are effective therapies for morbid obesity with high rates of T2DM resolution [2]. Until recently, however, bariatric surgery in the HIV-positive population remained controversial [3].

The first report of a patient with HIV infection undergoing bariatric surgery was in 2005 [4], and, subsequently, a small number of studies, including within our own unit, have reported outcomes [5]. Bariatric surgery is now considered a safe and effective treatment for people with morbid obesity who are also infected with HIV [6]. Notably, to date, there are limited descriptions of T2DM outcomes in such individuals. Given the increasing prevalence of this combination of conditions, we present a case series to advance this discussion.

## Cases presentation

### Methods of case collection

We studied 120 patients with T2DM who underwent bariatric surgery between 2010 and 2017 at Chelsea and Westminster Hospital, London. The patients groups were: AGB (*n* = 62) and SG (*n* = 58). Three patients known to be HIV antibody positive form the basis of this series. Selection for bariatric surgery was consistent with National Institute for Health and Care Excellence (NICE) guidelines with procedural type co-decided by the patient and the multidisciplinary team (MDT). Procedural descriptions are provided elsewhere [7]. Utilizing hospital pathology and electronic record systems, information was collected on: demographics; anthropometrics; weight history; surgical details; perioperative diabetes status; perioperative HIV status, and major outcomes.

### Case 1

Case 1 is a 58-year-old Caucasian male with a history of HIV infection (2002), T2DM (2008), and obesity. His comorbidities included hypertension, dyslipidemia, and obstructive sleep apnea. (Table 1). Preoperatively, he was prescribed metformin 500 mg twice a day and glycated hemoglobin (HbA1c) was 40 mmol/mol. His baseline body mass index (BMI) was 47 kg/m^2^, with a weight of 162.9 kg. Multiple attempts at weight loss, including commercial diets and orlistat, had been unsuccessful. HIV prescriptions included one tablet daily of Atripla (efavirenz/emtricitabine/tenofovir). His preoperative CD4 count was 800 cells/μL and viral load was undetectable. Following assessment by the bariatric MDT, he was found to meet criteria for surgery.

**Table 1:** Preoperative assessment for the bariatric surgery on admission

	Case 1	Case 2	Case 3
Past medical history	Hypertension, dyslipidemia, T2DM, obstructive sleep apnea, obesity, gout, Burkitt’s lymphoma, HIV, CKD stage 2, hydrocele repair, tonsillectomy	Obesity, T2DM, obstructive sleep apnea, depression, HIV, fatty liver disease, tonsillectomy	Obesity, T2DM, asthma, dyslipidemia, obstructive sleep apnea, urinary incontinence, peripheral neuropathy, knee osteoarthritis, depression, vitamin D deficiency.
Drug history	Allergic to co-trimoxazole. Metformin 500 mg twice a day Losartan 100 mg once a day Allopurinol 200 mg once a day Atorvastatin 10 mg once a day Indapamide 1.5 mg once a day	No known drug allergy. Mirtazapine 30 mg once a day Metformin 500 mg once a day	No known drug allergy. Ranitidine 300 mg once a day Atorvastatin 10 mg once a day Metformin 1 g three times a day Dapagliflozin 10 mg once a day Exenatide 20 mcg once a day Detemir 70 units twice a day
HIV medications	Atripla (efavirenz/emtricitabine/tenofovir) 1 tablet once a day	Atripla (efavirenz/emtricitabine/tenofovir) 1 tablet once a day	Truvada (emtricitabine/tenofovir) 245/200 mg once a day Darunavir 800 mg once a day Ritonavir 100 mg once a day
Family history	Father – aortic aneurysm Mother – Alzheimer’s	Nil	Nil
Social history			
Tobacco smoking	Nil	Occasional	Nil
Alcohol	40 units/month	Occasional	Nil
Employment	Computer programmer	Unemployed	Unemployed
Independence	Lives with family, independent of daily activities	Lives with friends, independent of daily activities	Lives with daughter, wheelchair bound most of the time
Observations	HR – 70	HR – 100	HR – 83
	RR – 16	RR – 16	RR – 18
	Sats – 99%	Sats – 95%	Sats – 95%
	BP – 128/72	BP – 136/90	BP – 145/83
	T – 36.4 °C	T – 36.1 °C	T – 36.7 °C
Physical examinations	Mild right knee joint pain	Mild bilateral joint pain and low back pain. Fungal infection right axilla	Mild bilateral joint pain and low back pain.
Neurology examinations	NAD	NAD	Numbness below the knee bilaterally. Urinary incontinence

*BP* blood pressure, *CKD* chronic kidney disease, *HIV* human immunodeficiency virus, *HR* heart rate, *NAD* no abnormality detected, *RR* respiration rate, *Sats* oxygen saturation, *T* temperature, *T2DM* type 2 diabetes mellitus

**Fig. 1:**
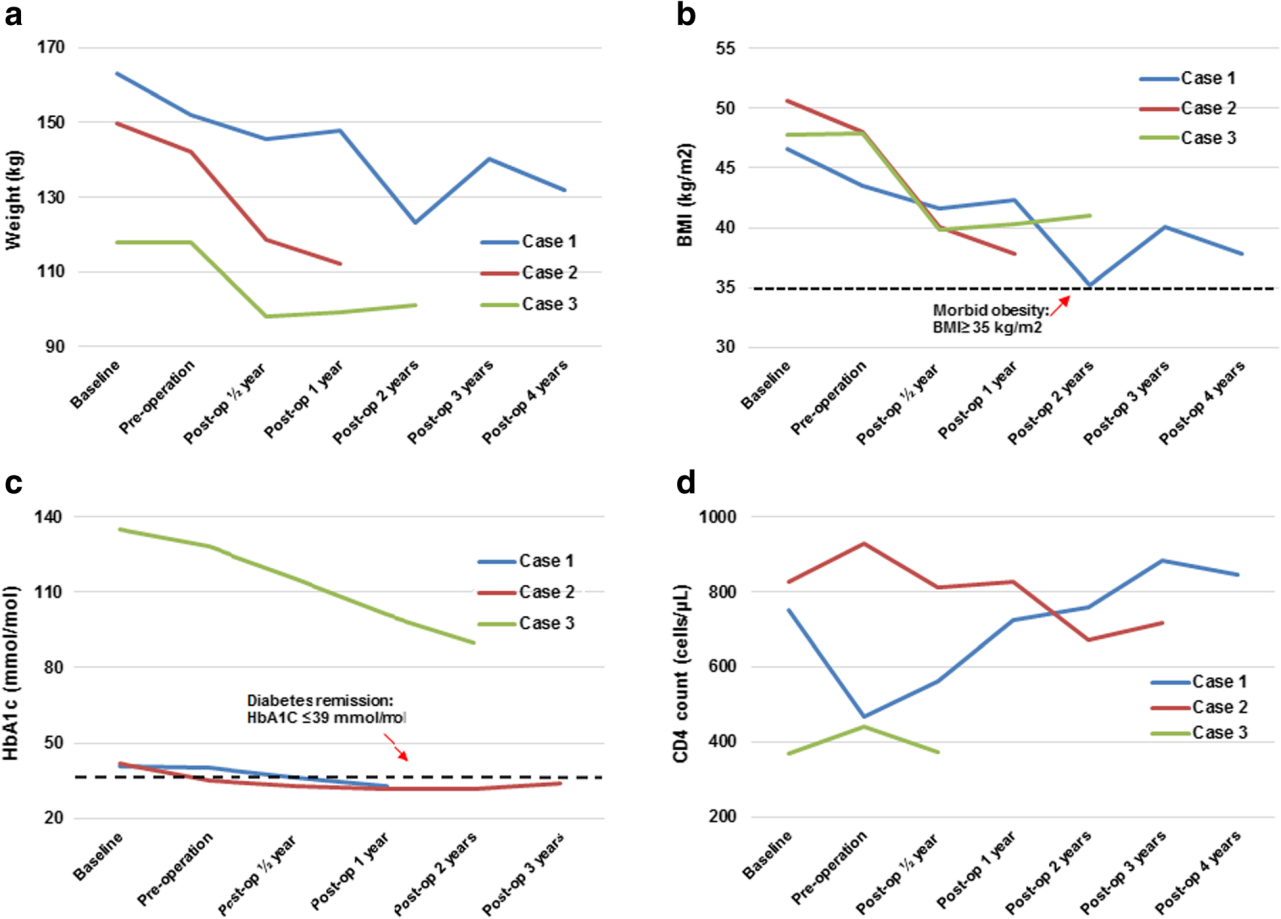
Line graph illustrating changes in clinical parameters for Cases 1–3. **a**, **b** Weight status. **c** Glycemic control. **d** Human immunodeficiency virus status. BMI body mass index, HbA1c glycated hemoglobin

In 2012 he underwent laparoscopic AGB surgery and had an uncomplicated postoperative course. Preoperative and postoperative clinical parameters are presented in Tables 1, 2, and 3 and Fig. 1 with sustained weight loss reported. As per local guidelines, this patient continued to receive metformin 500 mg twice a day postoperatively to optimize insulin sensitivity. Six months postoperatively, HbA1c was 35 mmol/mol, and there was no evidence of diabetes-related complications. His HIV infection status was not affected by surgery, and he continued to receive Atripla (efavirenz/emtricitabine/tenofovir). His CD4 count was unchanged at each postoperative visit, with undetectable viral load throughout. He continues to be on antiretroviral and antidiabetic medications as well (metformin 500 mg twice a day) and reports sustained weight loss.

**Table 2:** Preoperative and final postoperative clinical parameters for Cases 1–3

	Case 1		Case 2		Case 3	
	preoperatively	Postoperatively^4^	preoperatively	Postoperatively^4^	preoperatively	Postoperatively^4^
BMI (kg/m^2^)	44	37.8	48.1	37.9	47.9	41.1
Weight (kg)	152.1	132.1	142.2	112.0	118.0	101.2
% TWL^1^	6.6%	18.9%	5.1%	25.2%	−0.2%	14.1%
% EWL^2^	14.3%	40.8%	10.0%	49.8%	−0.4%	29.5%
HbA1c (mmol/mol)^3^	41	33	35	34	128	90
Diabetes medications	Metformin 500 mg twice a day	Metformin 500 mg twice a day	Metformin 500 mg once a day	Nil	Metformin 1 g three times a day Dapagliflozin 10 mg once a day Exenatide 20 mcg once a day Detemir 70 units twice a day	Metformin 1 g twice a day Dapagliflozin 10 mg once a day Humulin M3 (human insulin, mixture 3) (22 units OM, 16 units ON)
CD4 count (cells/μL)	750	845	929	718	440	372
Viral load (cp/ml)	< 40	< 40	< 40	< 20	< 40	< 20
HIV medications	Atripla 1 Tab once a day	Atripla (efavirenz/emtricitabine/tenofovir) 1 Tab once a day	Atripla (efavirenz/emtricitabine/tenofovir) 1 Tab once a day	Truvada (emtricitabine/tenofovir) 245/200 mg once a day Raltegravir 400 mg twice a day	Truvada (emtricitabine/tenofovir) 245/200 mg once a day Darunavir 800 mg once a day Ritonavir 100 mg once a day	Truvada (emtricitabine/tenofovir) 245/200 mg once a day Rezolsta (darunavir/cobicistat) 800/150 mg once a day
Complications		Vitamin D deficiency		Nil		Stricture

*BMI* body mass index, *EWL* excessive weight loss,* HbA1c* glycated hemoglobin, *HIV* human immunodeficiency virus, *Tab* tablet, *TWL* total weight loss, *OM* in the morning, *ON* at night. ^1^ % *TWL*: percentage of total weight loss, ^2^ % *EWL*: percentage of excess weight loss, calculated by dividing weight changes from baseline by excess body weight. The latter value was obtained by subtracting the ideal body weight as that equivalent to a body mass index of 25 kg/m2 from the actual baseline weight, ^3^ normal range of glycated haemoglobin is 20–41 mmol/mol, ^4^ last follow-up (> 3 years in all cases)

**Table 3:** Results of routine laboratory tests pre-bariatric operation and post-bariatric operation

	Case 1		Case 2		Case 3	
	Preoperation*	Postoperation^	Preoperation*	Postoperation^	Preoperation*	Postoperation^
FBC						
Hb (g/L)	129	156	143	165	122	119
WCC (×10^9^/L)	4.9	5.9	8.1	7.3	4.3	3.2
PLT (×10^9^/L)	149	204	186	243	270	182
CRP (mg/L)	28	5	1	12	22	1
U&Es						
Na (mmol/L)	138	142	138	139	137	144
K (mmol/L)	3.8	3.6	4.3	4.6	4.1	4.3
Urea (mmol/L)	4.6	6.5	2.2	5.4	4.0	7.2
Cr (mmol/L)	94	139	67	54	80	65
eGFR (ml/minute/1.73m^2^)	73	47	> 90	> 90	67	> 90
LFTs						
Bili (μmol/l)	10	6	9	9	6	4
ALP (IU/L)	30	34	45	30	10	19
ALT (IU/L)	59	65	80	95	109	66
ALB (g/L)	36	40	35	41	28	33
Urine analysis						
Specific gravity	1.030	1.030	1.015	1.020	1.030	1.010
pH	5.5	5.0	6.0	5.5	5.5	6.0
Protein (mg/L)	Negative	+	Negative	Negative	+	+
Glucose (mmol/L)	Negative	Negative	Negative	Negative	+	+
Urine culture	Negative	Negative	Negative	Negative	Negative	Negative
Fecal culture	N/A	N/A	N/A	N/A	N/A	N/A
Blood culture	N/A	N/A	N/A	N/A	N/A	N/A

*ALB* albumin, *ALP* alkaline phosphatase, *ALT* alanine aminotransferase, *Bili* bilirubin, *Cr* creatinine, *CRP* C-reactive protein, *eGFR* estimated glomerular filtration rate, *FBC* full blood count, *Hb* hemoglobin, *K* potassium, *LFTs* liver function tests, *Na* sodium, *N/A* not applicable, *PLT* platelet, *U&Es* urea and electrolytes, *WCC* white cell count

*on admission to receive bariatric surgery, ^last follow-up (> 3 years postoperation for all cases)

### Case 2

Case 2 is a 33-year-old Caucasian male who was positive for HIV (2011) with a background of T2DM, obesity, depression, and fatty liver disease (Table 1). His baseline BMI was 50.7 kg/m^2^ with a weight of 149.8 kg. Following 2 years of orlistat therapy and lifestyle intervention, his BMI decreased modestly to 48.1 kg/m^2^. Preoperatively, T2DM was controlled with metformin 500 mg once a day and his HbA1c was 35 mmol/mol. Following 2 years of HAART for which he received Atripla (efavirenz/emtricitabine/tenofovir) 1 tablet once a day, his CD4 count increased to 929 cells/μL from 552 cells/μL at diagnosis. Viral load was undetectable. Further preoperative and postoperative parameters are presented in Tables 1, 2, and 3 and Fig. 1.

A laparoscopic SG was performed in 2013. He reported no complications at postoperative follow-up. T2DM was diet controlled following surgery and his HbA1c remained stable (33 mmol/mol mean). Therefore, complete diabetes remission was achieved according to American Diabetes Association (ADA) criteria [8]. Postoperatively, his viral load remained undetectable with a mean CD4 count of 735 cells/μL. Following clinical trial recruitment, antiretroviral medication was adjusted in an attempt to better stabilize mood. Depressive symptoms improved and HIV status remained stable.

### Case 3

Case 3 is a 48-year-old Caucasian female with a history of obesity, HIV disease (2003), and poorly controlled T2DM with peripheral neuropathy (2003) (Table 1). Her baseline BMI was 47.8 kg/m^2^ and multiple attempts at weight loss had been unsuccessful. Her preoperative HIV status was well controlled (CD4 count 440 cells/μL, undetectable viral load) with Truvada (emtricitabine/tenofovir), darunavir, and ritonavir. Unfortunately, despite various treatments of sodium-glucose co-transporter-2 (SGLT-2) inhibitor, high-dose insulin sensitizer, glucagon-like peptide-1 (GLP-1) agonist, and high-dose basal insulin, her HbA1c remained elevated at 128 mmol/mol. Extensive discussions were undertaken with the patient and the MDT. Despite lack of glycemic optimization, benefits were deemed to outweigh risks and so SG was scheduled.

Preoperative and postoperative clinical parameters are presented in Tables 1, 2, and 3 and Fig. 1. Her T2DM status improved following surgery: HbA1c dropped to 90 mmol/mol 2 years postoperatively (accompanying fasting glucose of 12 mmol/L). Unsurprisingly, given T2DM duration, preceding control, and preoperative insulin requirements, diabetes remission was not achieved in this case. Following surgery, however, she benefits from a reduced pill burden and markedly reduced daily insulin requirements (38 versus 140 units preoperatively). Anti-retroviral medications were switched to Truvada (emtricitabine/tenofovir) and Rezolsta (darunavir/cobicistat) and her HIV status remained stable (CD4 count 400 cells/μL, undetectable viral load). An esophageal stricture which developed 2 years postoperatively responded to a dilatation procedure. No further complications have occurred.

## Discussion

Here we present three differing cases which add to the literature supporting bariatric surgery as a safe treatment modality in individuals who are HIV positive. Our cases series is novel as we have compared the effects of bariatric surgery on weight reduction and glycemic control in patients with HIV infection as well as patients without HIV infection.

T2DM prevalence and complication rates in the HIV-infected population (23–40%) are noticeably higher than the general population [9]. Traditional risk factors as well as HIV-specific factors including anti-retrovirals and lipodystrophy syndrome contribute to the pathogenesis [9]. A strong body of evidence supports the use of bariatric surgery as a treatment modality for T2DM in the context of obesity [10] with sustained remission of T2DM described [7]. Also reported are improvements in cardiovascular risk profile, obesity-related complications, and all-cause mortality [2, 7]. Despite this, bariatric surgery remains an underutilized tool and data are limited for the HIV-infected population. Although reports [11, 12] have suggested that bariatric surgery is safe, there is a paucity of data describing the outcomes of T2DM in these individuals or, in fact, the uptake of surgery.

Summarized clinical outcomes for our case series (*n* = 3) are presented alongside outcomes for patients with T2DM who were not HIV infected (*n* = 117) in Table 4. Case 1 (AGB) achieved weight loss, which was 53% excessive weight loss (EWL) in excess of the figure typically quoted for this procedure (40%) [13]. This is particularly impressive as a restrictive procedure. Cases 1 and 2 were also noted to achieve greater % EWL compared to the non-HIV group for their respective procedures. Case 3, however, achieved below average % EWL for SG (30% compared to 60% reported [14]), although this was not far from the average % EWL for the non-HIV group (42% ± 20%). We speculate that several patient factors, including negative eating habits, depression, and sedentary life style, may all have contributed to this outcome.

**Table 4:** Summary of clinical outcomes in patients who are human immunodeficiency virus positive and patients who are not human immunodeficiency virus positive referred for bariatric surgery

	Baseline BMI	%TWL postop after 2 years	%EWL postop after 2 years	HbA1c		Complete remission of T2DM^1^
				Preop	Postop after 1 year	
HIV case 1 (AGB)	46.6	24.4%	52.6%	41	33	No
HIV case 2 (SG)	50.7	25.2%	49.8%	35	32	Yes
HIV case 3 (SG)	47.8	14.1%	29.5%	128	90	No
AGB-non HIV (n = 61)	43.4 ± 6.2	14% ± 8%	34% ± 22%	60.6 ± 18.1	58.2 ± 13.5	5%
SG-non HIV (n = 56)	49.6 ± 10.7	22% ± 9%	42% ± 20%	59.5 ± 18.5	54.4 ± 18.1	27%

*AGB* adjustable gastric band, *BMI* body mass index, *EWL* excessive weight loss, *HbA1c* glycated hemoglobin, *HIV* human immunodeficiency virus, *SG* sleeve gastrectomy, *T2DM* type 2 diabetes mellitus, *TWL* total weight loss. ^1^ according to American Diabetes Association criteria [8]. Data in non-HIV group were described as mean ± standard deviation

In terms of T2DM, although only Case 2 achieved remission according to ADA criteria [8], it is notable that all cases achieved an improvement in HbA1c postoperatively. Ongoing monitoring for relapse is advisable. Case 1 would also have achieved remission were it not for the continuation of metformin postoperatively. Although T2DM outcomes for Case 3 did not objectively seem as successful, it is notable that individual insulin requirements and pill burden were reduced. Lack of remission was perhaps predictable given the longevity of T2DM and preoperative insulin dosage.

In all three cases, HIV status was not affected by bariatric surgery, which is consistent with existing literature [4, 6, 11, 12, 15]. There is a theoretical concern over drug absorption following bariatric surgery. One study to date has reported that, despite a mild reduction, drug levels following SG remained within the therapeutic range [12]. In our case series, the HIV status was not adversely affected by bariatric surgery.

## Conclusions

In conclusion, our case series further supports the use of bariatric surgery as a safe treatment modality in individuals who are HIV positive [11]. Importantly, we have demonstrated the positive effect of bariatric surgery on T2DM in this group of patients. Further work would be beneficial to consolidate these findings.

## Abbreviations

ADA: American Diabetes Association
AGB: Adjustable gastric banding
BMI: Body mass index
EWL: Excessive weight loss
GLP-1: Glucagon-like peptide-1
HAART: Highly active antiretroviral treatment
HbA1c: Glycated hemoglobin
HIV: Human immunodeficiency virus
MDT: Multidisciplinary team
NICE: National Institute for Health and Care Excellence
RYGB: Roux-en-Y gastric bypass
SG: Sleeve gastrectomy
SGLT-2: Sodium-glucose co-transporter-2
T2DM: Type 2 diabetes mellitus
